# Clinical Application Status of Articular Cartilage Regeneration Techniques: Tissue-Engineered Cartilage Brings New Hope

**DOI:** 10.1155/2020/5690252

**Published:** 2020-06-30

**Authors:** Shuangpeng Jiang, Weimin Guo, Guangzhao Tian, Xujiang Luo, Liqing Peng, Shuyun Liu, Xiang Sui, Quanyi Guo, Xu Li

**Affiliations:** ^1^Department of Orthopedics, The First Hospital of China Medical University, 155 Nanjing North Street, Heping District, Shenyang, 110001 Liaoning Province, China; ^2^Key Lab of Musculoskeletal Trauma & War Injuries, PLA, Beijing Key Lab of Regenerative Medicine in Orthopedics, Chinese PLA General Hospital, Beijing 100853, China

## Abstract

Hyaline articular cartilage lacks blood vessels, lymphatics, and nerves and is characterised by limited self-repair ability following injury. Traditional techniques of articular cartilage repair and regeneration all have certain limitations. The development of tissue engineering technology has brought hope to the regeneration of articular cartilage. The strategies of tissue-engineered articular cartilage can be divided into three types: “cell-scaffold construct,” cell-free, and scaffold-free. In “cell-scaffold construct” strategies, seed cells can be autologous chondrocytes or stem. Among them, some commercial products with autologous chondrocytes as seed cells, such as BioSeed®-C and CaReS®, have been put on the market and some products are undergoing clinical trials, such as NOVOCART® 3D. The stem cells are mainly pluripotent stem cells and mesenchymal stem cells from different sources. Cell-free strategies that indirectly utilize the repair and regeneration potential of stem cells have also been used in clinical settings, such as TruFit and MaioRegen. Finally, the scaffold-free strategy is also a new development direction, and the short-term repair results of related products, such as NOVOCART® 3D, are encouraging. In this paper, the commonly used techniques of articular cartilage regeneration in surgery are reviewed. By studying different strategies and different seed cells, the clinical application status of tissue-engineered articular cartilage is described in detail.

## 1. Introduction

Hyaline articular cartilage is a highly specialised connective tissue that lacks blood vessels, lymphatics, and nerves. Nutrients diffuse into the articular cartilage from the synovial fluid, thereby limiting its self-healing ability. Articular cartilage injury is common, and the incidence of cartilage defects in patients undergoing arthroscopy is as high as 61-63% [1]. Articular cartilage injury can further lead to osteoarthritis (OA), which mainly manifests as swelling, pain, and deformity of the knee, hip, spine, and hand joints, resulting in limited joint movement and seriously affecting patients' quality of life. Symptomatic knee OA affects 24% of the total population worldwide [2], and the incidence of OA among people over 60 years of age in the United States is as high as 38-47% [3, 4]. The large number of patients becomes a heavy burden for the medical system. In addition, OA is the second major cause of physical disability behind cerebrovascular diseases in China, which seriously reduces the social labor force and puts a very large economic burden on society and families. In developed countries, the medical cost of OA accounts for 1.0%-2.5% of the gross domestic product (GDP) [5].

Currently, commonly used techniques of articular cartilage regeneration include microfracture (MF), osteochondral autologous transplantation (OAT), osteochondral allograft transplantation (OCA), particulated articular cartilage implantation (PACI), and autologous chondrocyte implantation (ACI). These methods all have their own limitations, making the complete regeneration of hyaline cartilage tightly bound to surrounding normal cartilage difficult [6]. In recent years, tissue engineering technology has been considered the most promising method for regenerating articular cartilage. This article will introduce these traditional methods and the concept of tissue-engineered articular cartilage according to different strategies and seed cells.

## 2. Current Strategies for Articular Cartilage Regeneration

### 2.1. Traditional Surgical Regeneration Techniques

#### 2.1.1. Microfracture

MF is a typical representative of bone marrow stimulation procedures, first reported by Rodrigo in 1994 [7]. MF uses bone marrow mesenchymal stem cells (BMSCs) and growth factors to form fibrous blood clots at the cartilage defect, and these cells differentiate into fibrous cartilage tissue to repair the cartilage defect.

MF has the following characteristics: (1) it can repair small defects better than large defects [8, 9] and best repairs defect sizes of 1-2.5 cm^2^ [10]; (2) it repairs defects in young patients, especially those under 40 years old, better than those in aged patients [11]; and (3) it repairs femoral condyle cartilage defects better than those in other parts of the knee joint (tibial plateau, femoral pulley, patella, etc.) [11].

Some studies have shown that MF has a good effect on the repair of articular cartilage defects [12, 13]. However, a substantial amount of evidence shows that the long-term treatment effect of MF is poor. The regenerated tissue is mainly fibrocartilage, which has poor mechanical properties, and the cartilage phenotype is difficult to maintain for an extended period [11, 14]. In a follow-up of 29 patients with knee MF, only 57% maintained good performance according to the International Cartilage Repair Society (ICRS) score at the second arthroscopy performed 12.4 months after surgery, and biopsies showed that the regenerated tissue was fibrocartilage [15]. However, for the remaining 43%, the regenerated tissue was fibroelastic, which was significantly different from the surrounding normal articular cartilage.

MF has a definite short-term efficacy and costs less and is less difficult to perform than other methods, but its long-term treatment effect is uncertain. There is evidence that retrograde changes in the subchondral region, such as cysts, excessive bone growth, and osteophyte formation [1], may occur after MF, which will limit its use.

#### 2.1.2. Osteochondral Transplantation (OCT)

**Figure 1:**
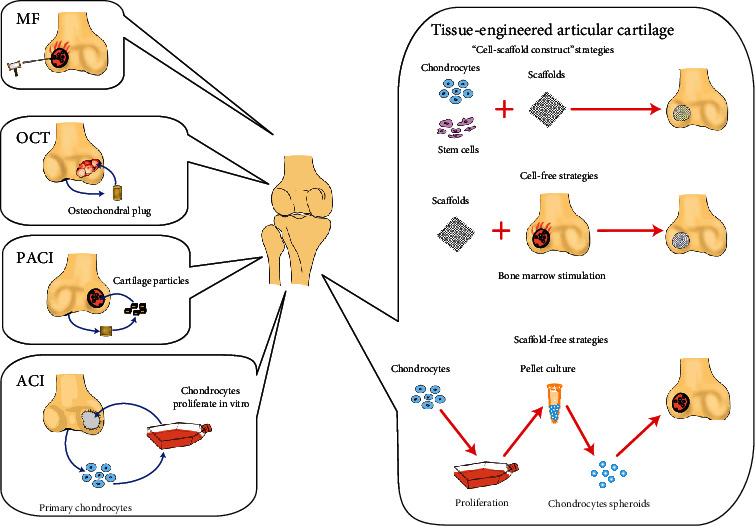
Articular cartilage regeneration techniques.

*(1) OAT*. OAT (Figure 1) refers to the removal of osteochondral columns from the patient's non-weight-bearing articular surfaces, such as the femoral trochlear, and their transplantation into repaired articular cartilage defects, known as mosaics. Yamashita et al. [16] first reported the use of autologous osteochondral slice transplantation for the treatment of knee cartilage defects in 1985.

OAT surgery has the advantages of being simple to perform, healing rapidly, and having no immune rejection. It can immediately fill a defect with hyaline cartilage; the surface of which is composed of independent osteochondral columns, though they are not truly bound to each other as a whole. OAT is suitable for patients with a small defect size or exfoliative osteochondritis [17]. Hangody et al. [18] followed 354 patients for 2 to 17 years, and the results showed that OAT had good to excellent effects on 91% of femoral lesions, 86% of tibial lesions, 74% of patella femoral lesions, and 92% of talus bone lesions. This is consistent with the findings of Shimozono et al. [19], whose meta-analysis showed that OAT could provide a good midterm treatment effect for patients with talus osteochondropathy.

The main limitations of OAT are secondary lesions at the donor site and limited sources of grafts, limiting its use to only small defects. A systematic review by Andrade et al. [20] showed that the average secondary lesion incidences at OAT donor sites in the knee and ankle were 5.9% and 19.6%, respectively. Therefore, the risk-benefit ratio should be carefully evaluated before clinical application of OAT. In addition, the autologous osteochondral column cannot perfectly match the shape and curvature of the defect area, which may cause stress concentration and long-term surgical failure.

*(2) OCA*. OCA (Figure 1) is a one-step reconstruction of the complete articular surface using allogeneic osteochondral tissue. Because of the low immunogenicity of articular cartilage, OCA is clinically applicable and does not require immunosuppression [21]. Compared with OAT, OCA has the advantages of causing less trauma, being simpler to perform, and having a wider graft source, thus avoiding the occurrence of donor site morbidity; furthermore, OCA can repair a large area of cartilage defects at one time and reconstruct the complete articular surface composed of mature hyaline cartilage. In addition, both fresh and frozen allografts can be used for OCA [22].

Krych et al. [23] confirmed that OCA treatment of knee cartilage and osteochondral defects can improve the recovery rate. Levy et al. [24] also showed that the 10-year survival rate was 82% after fresh OCA of the femoral condyle, and the patients' symptoms and functions continued to improve. Because of anatomical differences, the incidence of medial femoral condylar cartilage defects is approximately 6 times that of the lateral femoral condyle [25]. Mologne et al. [26] have proven that allogenic lateral femoral condyle transplantation can be used to repair medial femoral condyle cartilage defects, and there is no significant difference in the effect of repairing the medial femoral condyle with the medial femoral condyle graft, which expands the source of transplantation for medial femoral condyle OCA.

Serological and microbiological detection of fresh OCA plants takes approximately 14 days [27], but the activity of fresh OCA grafts under traditional preservation conditions can last only 7 days in vitro. In the 1970s, Mankin et al. [28] began to treat bone tumors with frozen human osteochondral allografts. Although freezing the osteochondral graft prolongs its storage time and further reduces its immunogenicity, it reduces the activity of chondrocytes and the cartilage matrix content [29], and the long-term repair effect is poor [30]. Some scholars have used “prolonged fresh” grafts to preserve fresh OCA grafts for 42 days, which may solve the problem of graft preservation [31].

In summary, allograft preservation, the risk of OCA disease transmission, graft failure, and high costs are the key problems for OCA.

#### 2.1.3. PACI

*(1) Autologous PACI*. PACI refers to crushing autologous or allogeneic articular cartilage into small particles sized 1-2 mm and then implanting them into articular cartilage defects. Studies have found that PACI is beneficial for chondrocyte migration and promotes cartilage regeneration [32]. The cartilage autologous implantation system (CAIS) of the Depuy company (USA) (Table 1) aims to implant autologous cartilage particles back into the cartilage defect area to regenerate articular cartilage. This autologous PACI has not been reported in large-scale clinical trials, but the repair effect in a phase I clinical trial was satisfactory [33]. The results showed that patients with CAIS had significantly better international knee documentation committee (IKDC) scores and knee injury and OA outcome scores (KOOS) than those in the MF group. In addition, the rate of osteophyte formation in the MF group was significantly higher than that in the CAIS group.

*(2) Allogeneic PACI*. Currently, there are commercial products derived from allogeneic juvenile cartilage particles, such as Zimmer's DeNovo NT system (Table 1), which has a retention period of up to 45 days in vitro. The repair effect is reportedly good within 2 years after implantation, and most of the repaired tissues are a mixture of hyaline cartilage and fibrocartilage [34]. Tompkins' retrospective study [35] included 15 patients treated with DeNovo NT with an average cartilage defect size of 2.4 cm^2^ and an average follow-up time of 28 months. MRI examination showed that cartilage repair was normal or nearly normal in 73% of patients, with an average filling rate of 89%.

Compared with OCT, PACI theoretically requires less donor cartilage and thus causes less damage to the donor. Unfortunately, the reported cartilage defects in both the CAIS and DeNovo NT system are small (<3.5 cm^2^) and lack long-term follow-up data. The safety and efficacy of the treatment have yet to be verified.

#### 2.1.4. Autologous Chondrocyte Implantation

ACI (Figure 1) involves the harvesting of chondrocytes from the non-weight-bearing area of the autologous articular surface, culture and amplification in vitro, and implantation into cartilage defects. Three generations have passed since Brittberg et al.'s first reported the repair of articular cartilage defects via ACI in 1994 [36]. In the first generation of ACI (P-ACI), the autologous periosteum was sutured onto the edge of the cartilage defect, and chondrocytes were injected into the defect lacunas. The second generation of ACI (C-ACI) involved the replacement of periosteal tissue with a type I/III collagen membrane. The third generation of ACI, namely, matrix-induced autologous chondrocyte implantation (MACI), belongs to the category of cartilage tissue engineering and will be discussed in the next section.

Compared with OAT, ACI has the advantages of causing less trauma and being simpler to perform, and compared with OCA, ACI has the advantages of no immune rejection and no risk of transmitted diseases, among others. ACI is currently considered a promising way to regenerate hyaline cartilage.

*(1) The First Generation of ACI*. Peterson et al. [37] reported the results of 2-9 years of follow-up of the first group of patients receiving P-ACI in 2000. The results showed that the clinical symptoms, arthroscopic examination, and histological results of the patients were significantly improved. In 2002, Peterson et al. [38] reported the durable effect of regenerated cartilage in 61 patients with ACI; although 5-11 years (average, 7.4 years) had passed, some patients still exhibited fibrocartilage-like repaired tissues. In addition, autogenous periosteum acquisition can cause secondary injury to patients. At the same time, some patients have periosteal hyperplasia, which usually requires arthroscopic resection [37]. These are problems to be considered in the clinical application of P-ACI.

*(2) The Second Generation of ACI*. To solve the problem of periosteal hypertrophy and iatrogenic trauma after P-ACI, C-ACI was performed. Steinwachs and Kreuz [39] followed up 63 patients with knee cartilage defects treated by ACI covered with a collagen I/III membrane for 3 years. The results showed that the ICRS and modified Cincinnati scores were significantly improved. There was no significant difference in the results at different defect sites, and the use of a collagen membrane prohibited graft hypertrophy. Rogers et al.'s study found that knee joint symptoms improved significantly 6 years after C-ACI treatment [40], which proved that C-ACI can provide long- and medium-term effects.

Although the above studies showed that autologous chondrocytes have potential in cartilage regeneration, the following shortcomings still limit the application of ACI.

- Invasive surgery is required to obtain chondrocytes
- The number of chondrocytes available from the donor site is limited
- The morbidity of the donor site
- Shedding of collagen/periosteal membranes leads to the loss of chondrocytes
- The in vitro expansion of chondrocytes is prone to dedifferentiation, and maintaining the chondrocyte phenotype is difficult [41]
- Autologous chondrocytes have reduced proliferation and differentiation potential in aged patients, which limits their application [42]
- Due to the generation of mechanically inferior fibrocartilage, joint replacement surgery is often unavoidable [43]

### 2.2. Tissue-Engineered Articular Cartilage

According to the statistics, 250,000 articular cartilage repair surgeries (including arthroplasty, MF, OCT, and ACI) are performed annually in the United States. However, the regenerated tissues cannot always maintain the phenotype of hyaline cartilage, fill the entire defect, and tightly integrate with the surrounding cartilage [44]. In recent years, the cross-cooperation of material science, biomechanics, biochemistry, and cell biology has substantially furthered the field of tissue engineering. Tissue-engineered cartilage is considered to regenerate hyaline cartilage-like tissue and fill the entire defect [44], which is the most promising solution for articular cartilage regeneration. Tissue-engineered cartilage is composed of seed cells, scaffolds, and growth factors, and seed cells have a high proliferation potential, strong differentiation ability, and low immunogenicity, laying the foundation for tissue engineering. Autologous chondrocytes were the first type of seed cell studied by scholars. However, in recent years, compared with other types of seed cells, mesenchymal stem cells (MSCs) have been well studied due to their strong proliferation and differentiation ability and low immunogenicity [45].  Table 2 shows the advantages and limitations of the use of common cell types for articular cartilage regeneration. Bioactive factors secreted by MSCs have also been shown to regulate the local microenvironment and promote the repair of damaged tissues [46]. On the other hand, various advanced scaffold materials provide suitable surfaces for the adhesion, proliferation, and differentiation of seed cells. At the same time, the scaffolds provided the cells with appropriate spatial structure and mechanical support before new cartilage is formed. A review of tissue-engineered articular cartilage by “cell-scaffold construct,” cell-free, and scaffold-free strategies is presented in the following section.

#### 2.2.1. “Cell-Scaffold Construct” Strategies

Tissue engineering techniques based on “cell-scaffold constructs” are the most commonly used strategy. In the field of articular cartilage regeneration, MACI (Figure 1) is a typical tissue-engineered articular cartilage technology based on the “cell-scaffold construct” strategy. In MACI, autologous chondrocytes are planted into a scaffold, and the chondrocyte-scaffold construct is then implanted into the cartilage defect. Similarly, “cell-scaffold constructs” based on MSCs, embryonic stem cells (ESCs), and induced pluripotent stem cells (iPSCs) can also be constructed. The scaffold materials are mainly biomaterials such as type I/III collagen matrix [47] and alginate-agarose hydrogel [48]. These biomaterials have the advantages of high biocompatibility and nontoxicity of degradation products. Initially, the scaffold provides a surface, three-dimensional space and support for seed cells. As new cartilage is generated, the scaffold is gradually degraded.

*(1) MACI*. First, autologous articular chondrocytes have a natural cartilage phenotype and, in theory, are the easiest to use among all cell types for the generation of cartilage tissue. Second, despite the problem of morbidity at the donor site, chondrocytes are obtained from non-weight-bearing region of the joint, which can minimize the impact on the weight-bearing function of the joint. Third, the use of autologous chondrocytes does not have the risk of disease transmission or immune rejection. Therefore, autologous chondrocytes theoretically have excellent application value.

The results of some preclinical and early clinical trials demonstrate that MACI is superior to early ACI and microfracture [49]. However, the prospective randomized controlled study conducted by Bartlett et al. [50] showed that the ICRS score for symptomatic knee cartilage defects one year after MACI was lower than that in the C-ACI group, as was the percentages of hyaline cartilage and hyaline cartilage with fibrocartilage (MACI group = 36.4% versus C − ACI group = 43.9%). Moreover, graft hypertrophy was not avoided in the MACI group (6%). Benthien et al. [51] systematically reviewed 6920 patients with knee cartilage defects, revealing that MACI was no better than ACI, MF, or OAT.

In recent years, numerous MACI commercial products have been developed, some of which are listed below:

1. BioSeed®-C (Table 1) is available in several European countries and has been used in more than 3,000 patients [44]. BioSeed®-C employs fibrin glue as the cell carrier to grow the cells on the polyglactin 910/poly-p-dioxanone fleece scaffold. Clinical studies (*n* = 79) have shown significant beneficial effects of BioSeed®-C therapy [52, 53]. KOOS scores were remarkably higher than those at baseline at 2 years of follow-up. Histological analysis showed good integration and formation of cartilage-repaired tissue. At 4 years, the ICRS, IKDC, KOOS, Lysholm, and Noyes scores of patients (n =50) were obviously improved. MRI showed a full fill of 72.7%, a moderate fill of 25%, and a less than 50% fill of 0.3% in patients.
2. Hyalograft® C (Table 1), which uses hyaluronic acid as a scaffold, has been used in more than 5,000 patients from 1999 to 2011 [54, 55]. A total of 28 studies (*n* = 793) reported clinical results showing that Hyalograft®C generally improves patient scores and, in some cases, is superior to microfracture treatment. Biopsy at different time points showed that hyaline cartilage accounted for 53%, fibrocartilage accounted for 22%, and mixed cartilage accounted for 25% of the total cartilage (*n* = 68) [44]. Unfortunately, there were no large-scale phase III clinical trials of the product, which has now been withdrawn from the market.
3. CaReS® (Table 1) is sold in some European countries, Turkey, Iran, and China. This product uses collagen type I hydrogels as scaffolds, but in particular, uses primary autologous chondrocytes as seed cells. A prospective multicenter study showed that the 36-item Short Form Survey (SF-36) and IKDC functional knee scores improved significantly from baseline. In addition, the total number of adverse events was significantly lower than that of ACI in patients at 30 months after CaReS® surgery [56]. However, the use of primary chondrocytes also disadvantageously results in a low implant cell density, which may influence the effect of cartilage regeneration.
4. NeoCart® (Table 1), another type I collagen scaffold [57], completed its phase III clinical trial in 2017. A 5-year follow-up of 29 patients treated with NeoCart® showed significant improvements in the magnetic resonance observation of cartilage repair tissue (MOCART) from 3 months to 24 months and a stable period from 24 months to 60 months. Furthermore, clinical patient-reported outcomes (PROs) were improved markedly compared with those at baseline [58]. However, 80% of patients developed subchondral bone lesion features, including edema, cysts, sclerosis, and hypertrophy, which may be related to the removal of the calcified cartilage layer before NeoCart implantation. Another 14% of the patients showed no improvement on MRI, suggesting that NeoCart® is not appropriate for all patients.
5. NOVOCART® 3D (Table 1) is a type I/III collagen biphasic scaffold. The trial by Müller et al. [59] confirmed that the IKDC score and visual analog scale (VAS) score at 6, 12, 24, and 36 months after NOVOCART® 3D implantation were significantly better than those preoperation. In addition, compared with the patients who received NOVOCART® 3D treatment after the failure of bone marrow stimulation, those who received NOVOCART® 3D as the first choice had significantly better postoperative IKDC and VAS results, and the operation failure rate was lower. Niethammer et al. [60] found that NOVOCART® 3D obviously improved postoperative IKDC and VAS scores in children and adolescents (<20 years) with articular cartilage defects compared to adults. The above research results show that NOVOCART®3D is an effective method for repairing articular cartilage defects, especially when used as the preferred treatment and for treating children and adolescents. Phase III clinical trials of NOVOCART® 3D are currently underway.

Most of these products require in vitro expansion of autologous chondrocytes. However, there is no unified standard for the time of in vitro proliferation and the cell passage used. Only CaReS® uses primary chondrocytes, but it inevitably faces the problems of low density and small numbers of seed cells. In addition, the scaffold materials used in each product are different but are mainly hydrogels, which can provide a microenvironment for only the growth and proliferation of cells and have inferior mechanical properties. In addition, although the short-term follow-up results of most products show that hyaline cartilaginous tissue can be regenerated, the longest follow-up period of the above products is only 5 years, and long-term follow-up data of large-scale randomized controlled trials (RCTs) are needed to prove the safety and effectiveness of various products. Finally, MACI inevitably faces limitations similar to those of ACI (see enumerations (a), (b), (c), (e), and (f) in Section 2.1.4).

*(2) “Cell-Scaffold Construct” Strategies Based on MSCs*. Professor Friedenstein [61] was the first to confirm the existence of MSCs in the bone marrow in 1968. In 2006, the international association of cell therapy (International Society for Cellular Therapy, ISCT) unified the definition of MSCs as follows: (1) adherent growth; (2) cell surface expression of certain specific antigens (markers); and (3) fat cell, osteoblast, and chondrocyte differentiation ability [62]. Since then, increasing evidence has shown that MSCs play an important role in tissue repair, regeneration, and immune regulation [63–66]. Now, the definition of MSCs has been extended, and the concept of “MSCs” as nonhematopoietic stem cells in the bone marrow was borrowed from the hypothesis of BMSCs but extended to nonskeletal tissue. BMSCs, including skeletal stem cells (SSCs), are believed to form and regulate local microvascular networks, regulate osteoclast differentiation, and establish and maintain the hematopoietic microenvironment necessary for growth and blood cell maturation. In addition, they may be necessary to maintain long-term self-renewing hematopoietic stem cells [67].

Several preclinical studies have shown that a single local (intra-articular) injection of autologous and allogeneic amplified MSCs can effectively reduce cartilage degeneration and joint inflammation in rats, pigs, and horses [68–71]. There are currently many types of MSCs with clinical applications, such as BMSCs and bone marrow-derived cells (BMDCs), adipose tissue-derived stem cells (ADSCs), peripheral blood progenitor cells (PBPCs) and peripheral blood-derived mesenchymal stem cells (PBMSCs), umbilical cord blood-derived mesenchymal stem cells (UCB-MSCs) and umbilical cord Wharton's jelly-derived mesenchymal stem cells (WJ-MSCs), and synovium/synovial fluid-derived mesenchymal stem cells(SMSCs/SFMSCs).

*(1) BMSCs and BMDCs (Figure 1)*. BMSCs have been obtained from a wide range of sources, have a strong in vitro expansion ability, and have the ability to differentiate into cartilage. Most importantly, BMSCs exist in the bone marrow cavity, and doctors can sometimes use BMSCs in tissue engineering strategies in conjunction with bone marrow stimulation. This method can achieve the goal of in situ cartilage regeneration in one step without the need for multiple operations. Therefore, BMSCs are the most commonly used MSCs in the field of articular cartilage regeneration.

Numerous preclinical studies and clinical trials have proven that BMSCs can repair articular cartilage defects [72, 73]. An observational cohort study showed that both BMSCs transplantation and P-ACI significantly improved patients' quality of life (the physiological and psychological parts of the SF 36 questionnaire), but the cost of one-knee surgery and the incidence of complications at the donor site were reduced in the BMSC group [74]. Wakitani et al. [75] implanted autologous BMSCs onto a collagen gel and transplanted them to articular cartilage defects. After 17-27 months of follow-up, clinical symptoms improved. The same results were observed in the trial performed by Haleem et al. [76], which showed that autologous BMSCs combined with platelet-rich fibrin glue scaffolds could significantly improve the symptoms of cartilage defects, and MRI examination showed that the defects were completely filled.

BMDCs are concentrates obtained from bone marrow and are generally considered to contain BMSCs [77]. Giannini et al. [78] used BMDC composite porcine collagen powder or hyaluronic acid membrane as a scaffold material to treat 48 patients with osteochondral lesions of the talus. After an average of 29 months (24-35 months), the American Association of Orthopedic Foot and Ankle Surgeons (AOFAS) score was increased remarkably, and histological evaluation showed different degrees of regenerative tissue remodeling. Although the histological examination did not show complete hyaline cartilage, this BMDCs strategy required only one operation, which reduced the pain and costs for patients compared with ACI or MACI.

However, bone marrow puncture wounds are still large, and researchers are looking for more minimally invasive and safe seed cell types.

*(2) ADSCs*. In recent years, ADSCs have been applied to tissue-engineered articular cartilage. Studies have shown that ADSCs have stronger immunoregulatory properties than BMSCs [79]. It is clear that compared to BMSCs, obtaining autologous ADSCs causes less trauma, has a lower risk of complications, and has a wider range of sources. A dose-escalation trial [80] showed that intra-articular injection of low-dose ADSCs could significantly alleviate knee pain caused by OA and improve joint function. The same results were also shown in a study by Koh et al. [81], which demonstrated that the effect of autologous ADSC-fibrin glue constructs combined with MF on OA was significantly better than that of MF alone. The radiological results and KOOS scores of patients in the ADSC group were distinctly improved. In the trial of Kim et al. [82], a commercially available Greenplast kit (Greencross, Seoul, Korea) was used as a scaffold to form a construct with ADSCs to treat OA. The 2-year follow-up results showed that ADSCs significantly improved the clinical symptoms of OA, the results of imaging evaluation were consistent with the clinical symptoms, and the articular cartilage defect was completely filled.

However, it cannot be ignored that postoperative histological assessments were not performed in any of the abovementioned experiments. It is not clear whether the filling tissue is comprised of hyaline cartilage or fibrocartilage, and the potential of ADSCs for osteogenesis and chondrogenic differentiation has been shown to be inferior to that of BMSCs [83]. Therefore, long-term follow-up results are needed to confirm the durability of the regenerated tissue.

*(3) PBPCs and PBMSCs (Figure 1)*. PBPCs and PBMSCs are convenient and minimally invasive materials that serve as good seed cells in tissue engineering. Numerous studies have demonstrated the effectiveness of PBPCs and PBMSCs in repairing nerve [84] and bone tissue [85]. In recent years, PBPCs and PBMSCs have been proven to be ideal seed cell choices in cartilage tissue engineering. In a clinical study, 180 patients with grade III-IV cartilage injury of the knee joint underwent subchondral bone drilling under arthroscopy [86]. After 5 weeks of continuous intra-articular injection of PBPCs and a hyaluronic acid mixture, secondary arthroscopy confirmed articular cartilage regeneration. Moreover, the histological examination showed hyaluronic cartilage characteristics. Subsequently, the RCT conducted by Saw et al. [87] compared the cartilage repair effect of an intra-articular injection of hyaluronic acid and hyaluronic acid combined with PBMSCs after subchondral bone drilling of the knee joint. Histological and MRI scores showed that the repair effect of hyaluronic acid combined with PBMSCs was significantly better than that of hyaluronic acid alone. In another case report [88], researchers treated a large full-thickness cartilage defect of the knee joint by activating autologous PBMSCs and autologous periosteal flap grafts and correcting patella joint dislocation. A follow-up of 7.5 years showed that the patients' exercise level returned to the preinjury level. The IKDC 2000 subjective score, Lysholm score, and Tegner score were 95, 98, and 9, respectively. CT and MRI evaluations showed significant improvement compared with those at the preoperative evaluation.

All of the above studies have proven that PBPCs and PBMSCs are optional seed cells for tissue-engineered cartilage. However, it is generally believed that PBPC and PBMSC numbers in peripheral blood are low, which limits their clinical applications. The key to ameliorating the insufficient number of PBPCs and PBMSCs lies in finding a better method for stem cell mobilization. Researchers have revealed that the traditional mobilization drug granulocyte colony stimulating factor (GCSF) has a poor effect on the mobilization of PBMSCs, and multidrug joint mobilization may be used in the future.

*(4) UCB-MSCs and WJ-MSCs (Figure 1)*. The use of umbilical cord-derived stem cells is completely noninvasive for patients. Compared with those of other adult stem cell types, the phenotype of umbilical cord-derived stem cells is more primitive, and their proliferation and differentiation abilities are stronger [89]. Compared with ESCs, umbilical cord stem cells pose no ethical problems. These advantages make umbilical cord-derived stem cells one of the most promising seed cells in tissue engineering regenerative medicine.

Park et al. [90] followed up on the ability of UCB-MSCs and a hyaluronic acid hydrogel composite to repair articular cartilage in OA patients for 7 years. Mature prosthetic tissue was observed by arthroscopy at 12 weeks after the operation, and the VAS and IKDC scores were improved at 24 weeks. Histological evaluations at one year revealed hyaline cartilage-like tissue, while the three-year MRI evaluation showed persistence of regenerated cartilage, and no osteogenesis or tumorigenesis occurred within seven years. The results of long-term follow-up support that UCB-MSCs are safe and effective seed cells for articular cartilage regeneration.

Histological examination confirmed that umbilical Wharton's jelly contains a large amount of type II collagen and proteoglycan and is thus very similar to the composition of articular cartilage. Therefore, WJ-MSCs have become a research hotspot in the field of articular cartilage regeneration. The low expression of HLA-I antigens in WJ-MSCs makes them less immunogenic and allows clinical allotransplantation without causing host immune rejection [91]. In addition, WJ-MSCs do not undergo malignant transformation, which is an important feature of clinical safety [92].

Preclinical studies have confirmed that WJ-MSCs can repair articular cartilage defects in animals [93]. However, there are few reports on the clinical application of WJ-MSCs. Boguslaw Sadlik implanted collagen scaffolds seeded with WJ-MSCs into the cartilage defect area of the knee joint through knee arthroscopy. MRI examination showed that the regenerated tissue was well integrated with the surrounding natural articular cartilage 9 months after the operation [89]. Unfortunately, no large-scale clinical application or long-term follow-ups have been reported.

*(5) SMSCs and SFMSCs (Figure 1)*. Previous studies have confirmed that SMSCs have greater chondrogenic potential than BMSCs, ADSCs, periosteum-derived MSCs, and muscle-derived MSCs [94, 95]. Unfortunately, there are few clinical reports on the use of SMSCs to repair articular cartilage defects. A small sample study by Sekiya et al. [96] reported 10 patients who received SMSC transplants. Four of these patients underwent a second arthroscopy, and the results showed that hyaline cartilage was regenerated in the cartilage defects in three patients, and fibrocartilage was filled in one patient. MSCs were also found in synovial fluid (SFMSCs). SFMSCs may be derived from MSCs shedding from synovial membranes, and genetic analysis showed that SFMSCs are very similar to SMSCs [97]. Jia et al. [98] showed that SFMSC-chitosan-based hydrogel constructs can repair rabbit full-thickness cartilage defects, and the regeneration tissue has typical hyaline cartilage properties. The results of previous studies based on SMSCs and SFMSCs are encouraging, and large-scale clinical trials are needed to confirm the safety and effectiveness of SMSCs and SFMSCs in repairing articular cartilage.

Although most of the above MSC-based studies provide hope for articular cartilage regeneration, the following problems need to be resolved for successful clinical application of MSCs:

- High variability of cartilage differentiation potential of MSCs in different individuals, taking BMSCs as an example: the biological characteristics of BMSCs are closely related to donor age and disease status. The proliferation and differentiation potential of BMSCs derived from aging individuals/advanced OA patients are significantly lower than those of young/healthy individuals, which largely limits the application of autologous BMSCs [99, 100]
- Harvesting MSCs from autologous bone marrow or adipose tissue has theoretical risks to the morbidity and infection of donor sites, while allogeneic MSCs may cause disease transmission and immune rejection [101]
- The properties of regenerated cartilage tissue are contradictory in different studies. Some results show that MSCs can regenerate only fibrocartilage with poor mechanical properties [102]
- Chondrocytes derived from MSCs have difficulty maintaining their phenotypes but tend to undergo hypertrophic differentiation, which leads to apoptosis and ossification [103]
- Some types of MSCs are scarce. As an example, BMSCs are a rare population, with a frequency of 0.01–0.001% [104].

*(3) “Cell-Scaffold Construct” Strategies Based on ESCs*. Embryonic stem cells are derived from the inner cell mass of the blastocyst and have the capacity for self-renewal and multidirectional differentiation. ESCs are totipotent stem cells and can be differentiated into any kind of cell. In theory, ESCs are ideal seed cells in the field of regenerative medicine. Currently, scientists have successfully induced the differentiation of ESCs into retinal pigment epithelial cells, neuronal cells, and cardiomyocytes by adding cytokines or other small molecules, and some clinical trials have used these ESC-derived cells to treat related diseases [105–107]. However, no clinical trials have been reported on the use of ESCs to repair human articular cartilage defects. Preclinical studies by Dattena et al. [108] confirmed that sheep ESC-fibrin glue constructs can promote the repair of sheep knee cartilage. The ESC group showed a better repair effect, but the difference from the control group was not significantly different, which may be related to the small number of cells used (500,000-700,000 cells/construct). Toh et al. [109] used a construct consisting of ESC-derived chondrocytes and hyaluronic acid-based hydrogels to repair osteochondral defects in the knee joints of rats and reported exciting experimental results. The repaired tissue had obvious hyaline cartilage characteristics, proving that hESC-derived chondrocytes are a potential source of seed cells for tissue-engineered articular cartilage.

The transformation of ESC-related preclinical research results into clinical practice faces the following problems:

- Ethical controversy, which is also the largest problem hindering the clinical application of ESCs
- Risk of disease transmission and immune rejection
- Tumorigenicity

*(4) “Cell-Scaffold Construct” Strategies Based on iPSCs*. IPSCs have self-renewal ability and multigerm layer differentiation potential and are obtained by inducing the reprogramming of differentiated adult cells. In 2006, four transcription factors, OCT4, SOX2, MYC, and KLF4, were selected by Takahashi and Yamanaka [110] and overexpressed in mouse fibroblasts by retroviral vectors to establish mouse iPSCs. For more than a decade, scientists have applied iPSCs to the construction of disease models, drug development, and regenerative medicine. Compared with ESCs, iPSCs have no ethical controversy, and they can be generated by reprogramming the patient's own cells, making autologous cell transplantation possible. Gratifyingly, differentiated cells produced by iPSCs exhibit young rather than adult properties, including faster proliferation and production of healthier and more durable repair tissues such as cartilage [111].

Preclinical studies have shown that iPSCs have great application value in the field of articular cartilage regeneration. Craft et al. [112] showed that in human pluripotent stem cell- (hPSC-) derived chondrogenic progenitor cells, activation of the TGF-*β* pathway promotes the effective development of articular chondrocytes, thereby forming stable cartilage tissue in vivo and in vitro. Saito et al. [113] showed that cartilage-differentiated human iPSCs (hiPSCs) can repair knee cartilage in mice, but some mice develop immature teratomas. Although there are no reports on the clinical application of iPSCs in repairing articular cartilage, their superior properties suggest that they would be effective in repairing articular cartilage.

However, we must be aware of the following iPSC shortcomings, which hinder their transformation to clinical applications:

- The preparation procedures are complicated and the technical requirements are high
- The preparation cost is expensive, which increases the financial burden on patients
- Reprogramming efficiency still needs to be improved
- The problem of tumorigenicity has not been resolved

#### 2.2.2. Cell-Free Strategies

Cell-free strategies are not absolutely “cell-free” and usually use endogenous stem cells indirectly. Cell-free tissue-engineered articular cartilage based on MSCs is an emerging concept that refers to the strategy of using MSC or MSC derivatives for cartilage regeneration without direct transplantation of MSCs. It can be divided into two categories: first, cartilage regeneration can be induced by in situ endogenous BMSCs using synthetic degradable scaffolds combined with bone marrow stimulation (Figure 1); second, the derivatives secreted by MSCs (such as extracellular vesicles, cytokines, and various RNAs) and other materials can be used to build composite scaffolds for implantation into cartilage defect areas for cartilage regeneration and repair. However, this kind of cell-free tissue-engineered articular cartilage is still in the animal test stage. Combined with bone marrow stimulation for the treatment of full-thickness cartilage defects or osteochondral defects, the “one-step method” can be utilized to complete the operation. At the same time, this method avoids the problems of iatrogenic trauma resulting from the harvesting of autologous chondrocytes or MSCs and the time and expense of cell culture and expansion in vitro. Currently, the representative products are TruFit scaffolds and MaioRegen scaffolds.

1. TruFit scaffold (Table 1).

The TruFit scaffold is composed of a polylactide-coglycolide copolymer, 10% calcium sulfate, polyglycolide fibers, and surfactant. During implantation, it is necessary to clear the subchondral bone in the cartilage defect area. Several early clinical studies with small samples showed that the TruFit scaffold could regenerate only fibrocartilage tissue [114, 115]. A recent clinical study with 2 years of follow-up also showed that the postoperative IKDCs score of patients who received the TruFit scaffold to repair cartilage defects were not significantly improved [116].

It is worth noting that clinical studies have shown that the TruFit scaffold repair of articular cartilage defects has a good long-term effect [117, 118], with improved clinical symptoms and radiological results. However, these studies did not compare TruFit scaffolds with traditional surgical cartilage repair techniques, and their conclusions need to be validated by large-scale randomized controlled studies. Currently, there are few comparative studies in this area. Hindle et al. [119] showed that the KOOS scores, EuroQoL Quality of Life Scale (EQ-5D) scores, and recovery abilities of patients with articular cartilage defects repaired by the TruFit scaffold were worse than those of the OAT group.

(2) MaioRegen scaffold (Table 1).

The MaioRegen scaffold is a three-layer biomimetic scaffold consisting of collagen I and hydroxyapatite. Studies have shown that it has a reliable medium-term effect on repairing articular cartilage defects [120]. A multicenter prospective study involving 49 patients with full-layer cartilage defects of the knee showed that MaioRegen scaffold implantation significantly improved knee symptoms, with significant improvements in the patients' average IKDC, VAS, and Tegner scores. MaioRegen scaffolds showed better efficacy in patients with exfoliative osteochondrositis and athletes [121], and a statistically significant correlation was observed between age and subjective IKDC scores at the 2-year follow-up. This result may have been due to the fact that the BMSCs of young patients have a stronger regenerative potential than those of older patients. Unfortunately, only 5 patients in this study underwent secondary arthroscopy, 4 of which exhibited failed repair. Histological examination showed that the central area of the regenerated tissue was fibrous.

Biomaterial scaffolds in combination with bone marrow stimulation infiltrate scaffolds with autologous bone marrow cytokines and BMSCs to induce cartilage regeneration in situ. However, few products are currently on the market, the number of application cases is small, and the clinical evaluations have mixed results. Large-scale clinical trials and long-term follow-up data are needed to prove effectiveness and safety. In addition, removal of the subchondral bone before implantation of the scaffold makes defect repair more difficult. The absence of subchondral bone requires that biomaterial scaffolds have the ability to regenerate both cartilage and subchondral bone and to ensure the integration of the two regenerative tissues.

#### 2.2.3. Scaffold-Free Strategies

The ideal scaffold of tissue-engineered articular cartilage needs to be suitable for cell adhesion and growth and to promote the secretion of extracellular matrix. The scaffold also needs to be gradually degraded according to the speed of cartilage regeneration to maintain the shape and mechanical strength of the cell-scaffold construct [122]. Unfortunately, no such ideal scaffold has been reported. Based on these reasons, interests in developing scaffold-free tissue-engineered articular cartilage (Figure 1) are high, but most studies are still in the preclinical experimental stage [122–124]. A few scaffold-free products are currently being used in clinical applications, and the representative product is Chondrosphere® (spherox) (Table 1).

Chondrosphere® comprises spheroids in suspension developed from autologous chondrocytes. Chondrocytes are cultured in vitro for 6-8 weeks to proliferate and concentrate into spheroids, which are then implanted into the defects. Co. Don's [125] Phase II RCT used Chondrosphere® to repair large articular cartilage defects (4-10 cm^2^) in patients, and the key result was that the KOOS score increased from baseline for 24 months, and the improvement at 24 months continued for 4 years. However, this trial did not establish a group without Chondrosphere®, thus limiting its value.

Then, a phase III clinical trial called COWISI, a key trial for the approval of Chondrosphere®, was performed [126]. To compare Chondrosphere® with MF, 102 patients with defect sizes of 1-4 cm^2^ were included in the trial. The National Institute for Health and Clinical Excellence (NICE) appraisal committee evaluated the results in October 2017 and concluded that Chondrosphere® was at least as effective in patients with small lesions as MF, while in patients with large lesions, Chondrosphere® improved outcomes for up to 4 years compared to the baseline parameters. The NICE recommends the use of Chondrosphere® for the treatment of femoral condyle and patellar cartilage defects only when (1) no previous repair of articular cartilage has been performed, (2) osteoarthritic damage is minimal; and (3) the area of cartilage defects exceeds 2 cm^2^ [126].

The scaffold-free strategy can be considered an ACI, but chondrocytes are no longer used in the form of cell suspensions in this strategy but are rather prepared into spheroids. This strategy avoids scaffold-related problems, preserves chondrocyte phenotypes, and provides a natural matrix component. Compared with cell suspension, scaffold-free tissue-engineered cartilage can reduce the loss of chondrocytes and maintain the defect for a long time. However, the strategy also faces the problems of requiring a long culture time and having a complex culturing procedure, which leads to increase of costs.

## 3. Future Prospects

Tissue-engineered cartilage is considered to be the most promising strategy for the complete regeneration of hyaline cartilage. Unfortunately, an optimal seed cell and scaffold material has not yet been found. Many scholars regard MSCs as ideal seed cells for tissue-engineered articular cartilage. However, the current clinical application of MSC-based tissue-engineered articular cartilage lacks long-term follow-up reports on large-scales. The possible immune rejection and tumorigenicity of MSC transplantation, as well as the obvious risk of disease transmission, cannot be ignored [127, 128].

In recent years, the field of regenerative medicine has furthered the understanding of the mechanisms of MSCs in repair and regeneration. A report indicates that few stem cells survive or remain in situ after injection [129]. At the same time, there is strong evidence that the therapeutic efficacy of stem cells is attributed to their paracrine effects rather than their direct differentiation [130]. By transplanting MSC derivatives (such as extracellular vesicles, cytokines and various RNAs) instead of directly transplanting MSCs, retaining the repair and regeneration function of MSCs has become an emerging research direction [131], and exosomes are attracting attention. Exosomes are small vesicles secreted by cells and are the main factors responsible for the biological functions of MSCs [132, 133]. Moreover, exosomes do not carry the immunogenicity and tumorigenic risk of MSCs and are thus ideal substitutes for MSCs in the field of tissue engineering. Numerous studies have confirmed that exosomes secreted by MSCs from different tissue sources can repair articular cartilage defects in animals [134, 135], but no clinical applications have been reported thus far.

On the other hand, iPSCs have been elucidated as a new cell source for regenerative medicine. The ethical problems that have long plagued ESCs have been resolved instantly, and technical problems in regard to iPSCs are gradually being resolved. At the same time, iPSCs also provide a source of cells for MSCs, and their unique superiority suggests that they have the ability to repair articular cartilage defects.

In the focus on scaffolds and cells, we must understand that cell-free strategies are not absolutely cell-free, but that scaffold-free strategies truly involve no scaffolds. The strategy selected should be tailored to the patient's specific circumstances, as each strategy has different advantages and disadvantages.

Cell-free strategies based on iPSCs or MSC derivatives may be a potential developmental direction for clinically addressing articular cartilage regeneration. The current challenges and possible solutions in this area are as follows:

1. Finding safe, effective, noninvasive, and ethically permissible types of cells: umbilical cord-derived MSCs may be the optimal choice
2. Finding the appropriate scaffold materials: the ideal scaffolds should have the biomimetic component and structure. The material closest to the natural articular cartilage may be the cartilage ECM
3. Finding the best cell derivatives: extracellular vesicles may be the most ideal MSC derivatives for tissue-engineered articular cartilage
4. To explore better cell (or cell derivative) scaffold-binding methods: freeze drying, hydrogel loading, and 3D bioprinting are possible developmental directions
5. To elucidate the molecular mechanism underlying the regeneration of articular cartilage by MSCs or their derivatives

Finally, basic research needs to be actively transformed into clinical applications, providing strong support for the clinical treatment of articular cartilage defects and OA.

## Figures and Tables

**Table 1:** Representative tissue-engineered articular cartilage products.

Classification	Product	Company	Application status	Seed cells	References
PACI	CAIS	Depuy (USA)	Phase I clinical trial has been completed	Autologous cartilage particles	[33]
	DeNovo NT	Zimmer (USA)	On the market	Allogenic juvenile cartilage particles	[34, 35]

“Cell-scaffold constructs” strategies	Bioseed®-C	BioTissue (Germany)	On the market in some European countries with more than 3,000 clinical applications.	Autologous chondrocytes (expansion in vitro)	[44, 52, 53],
	Hyalograft® C	Fidia Advanced Biopolymers (Italy)	Off the market, clinical applications exceeding 5,000	Autologous chondrocytes (expansion in vitro)	[44, 54, 55]
	CaReS®	Arthro Kinetics Biotechnology GmbH (Austria)	On the market in some European countries, Turkey, Iran, and China.	Autologous chondrocytes (primary)	[56]
	NeoCart®	Histogenics (USA)	Phase III clinical trial has been completed	Autologous chondrocytes (expansion in vitro)	[57, 58]

Cell-free strategies	TruFit	Smith & Nephew (USA)	On the market	/	[114–119]
	MaioRegen	Fin-Ceramica Faenza SpA (Italy)	On the market	/	[120, 121]

Scaffold-free strategies	Chondrosphere®(spherox)	Co.Don AG (Germany)	On the market	Autologous chondrocytes (spheroids)	[125, 126]

**Table 2:** Advantages and limitations of the use of common cell types for articular cartilage regeneration.

Cell types		Advantages	Limitations
Autologous chondrocytes		(1) Naturally have a chondrocyte phenotype (2) No immune rejection (3) No disease transmission	(1) Limited number of cells (2) The morbidity of the donor site (3) Chondrocytes dedifferentiation during expansion (4) Requires multiple surgeries (5) Regenerate fibrocartilage (6) Proliferation and differentiation potential decreased with age

MSCs	BMSCs/BMDCs	(1) Potential for chondrogenic differentiation (2) Theoretically has unlimited self-renewal ability (3) Can induce cartilage regeneration in situ	(1) Invasive surgery is needed for the harvesting (2) Low cell content (3) The morbidity of the donor site (4) Tumorigenicity (5) High variability in the chondrogenic differentiation potential of MSCs from different individuals (6) The differentiated chondrocytes are prone to hypertrophic differentiation
	ADSCs	(1) Abundant sources (2) Large number of cells (3) Good immunomodulatory capacity (4) Small invasive procedures to acquire cells	(1) Inferior potential for chondrogenesis (2) Tumorigenicity (3) High variability in the chondrogenic differentiation potential of MSCs from different individuals (4) The differentiated chondrocytes are prone to hypertrophic differentiation
	PBPCs/PBMSCs	(1) Cells harvested with minimal trauma (2) Low morbidity at the donor site	(1) Extremely low cell density (2) Stem cell mobilization is needed, and the procedure is complex (3) Tumorigenicity (4) High variability in the chondrogenic differentiation potential of MSCs from different individuals
	UCB-MSCs/WJ-MSCs	(1) Cells harvested noninvasively (2) Potential for chondrogenic differentiation (3) Ability for unlimited self-renewal under controlled conditions (4) Resistant to senescence	(1) Tumorigenicity (2) Risk of disease transmission (3) Lack of evidence from large-scale clinical trials
	SMSCs/SFMSCs	(1) Small invasive procedures to acquire cells (2) Potential for chondrogenic differentiation	(1) Tumorigenicity (2) Limited number of cells (3) Lack of evidence from large-scale clinical trials

ESCs		(1) Ability for unlimited self-renewal under controlled conditions (2) Capacity to differentiate into all mature cell types of the three germ layers, including chondrocytes	(1) Tumorigenicity (2) Risk of disease transmission (3) Immune rejection (4) Ethical controversy

iPSCs		(1) Capacity to differentiate into all mature cell types of the three germ layers, including chondrocytes (2) Ability for unlimited self-renewal under controlled conditions (3) No ethical controversy (4) Can be obtained from autologous adult cells	(1) Complicated preparation procedures and high technical requirements (2) High cost (3) Reprogramming efficiency still needs to be improved (4) Tumorigenicity
